# Trends and equity in the use of health services in Spain and Germany around austerity in Europe

**DOI:** 10.1186/s12939-021-01459-6

**Published:** 2021-05-13

**Authors:** Almudena Moreno, Lourdes Lostao, Johannes Beller, Stefanie Sperlich, Elena Ronda, Siegfried Geyer, José Pulido, Enrique Regidor

**Affiliations:** 1 Department of Sociology, Universidad Pública de Navarra, Campus de Arrosadía s/n, 31006 Pamplona, Spain; 2 I-COMMUNITAS - Institute for Advanced Social Research, Universidad Pública de Navarra, Pamplona, Spain; 3 Medical Sociology Unit, Hannover Medical School, Hannover, Germany; 4 Preventive Medicine and Public Health Unit, Universidad de Alicante, Alicante, Spain; 5 CIBER Epidemiología y Salud Pública (CIBERESP), Madrid, Spain; 6 Department of Preventive Medicine and Public Health, Universidad Complutense de Madrid, Madrid, Spain; 7 Instituto de Investigación Sanitaria del Hospital Clínico San Carlos (IdISSC), Madrid, Spain

**Keywords:** Austerity, Physician visits, Hospitalization, Education, Equity, Spain, Germany

## Abstract

**Background:**

Following the 2008 economic crisis many countries implemented austerity policies, including reducing public spending on health services. This paper evaluates the trends and equity in the use of health services during and after that period in Spain – a country with austerity policies – and in Germany – a country without restriction on healthcare spending.

**Methods:**

Data from several National Surveys in Spain and several waves of the Socio-Economic Panel in Germany, carried out between 2009 and 2017, were used. The dependent variables were number of doctor’s consultations and whether or not a hospital admission occurred. The measure of socioeconomic position was education. In each year, the estimates were made for people with and without pre-existing health problems. First, the average number of doctor’s consultations and the percentage of respondents who had had been hospitalized were calculated. Second, the relationship between education and use of those health services was estimated by calculating the difference in consultations using covariance analysis – in the case of number of consultations – and by calculating the percentage ratio using binomial regression – in the case of hospitalization.

**Results:**

The annual mean number of consultations went down in both countries. In Spain the average was 14.2 in 2009 and 10.4 in 2017 for patients with chronic conditions; 16.6 and 13.5 for those with a mental illness; and 6.4 and 5.9 for those without a defined illness. In Germany, the averages were 13.8 (2009) and 12.9 (2017) for the chronic group; 21.1 and 17.0 for mental illness; and 8.7 and 7.5 with no defined illness. The hospitalization frequency also decreased in both countries. The majority of the analyses presented no significant differences in relation to education.

**Conclusion:**

In both Spain and Germany, service use decreased between 2009 and 2017. In the first few years, this reduction coincided with a period of austerity in Spain. In general, we did not find socioeconomic differences in health service use.

## Introduction

During the economic crisis of 2008, the governments of a number of countries implemented austerity policies in order to deal with the crisis and the increase in public debt. Austerity policies are economic policies that aim to reduce the government’s budget deficit by reducing public spending, increasing taxes, or a combination of both. The measures implemented by many governments during the 2008 economic crisis were both: reductions in public spending and increases in taxes [1, 2]. One of the measures implemented was a reduction in spending on the health service, which consequently reduced staff numbers and physical resources in the system. Other measures included increasing the proportion of medication costs paid by patients and restrictions on some services [1]. These kinds of measures could lead to a decrease in health service use by some parts of the population. In addition, mental health problems are more frequent in subjects in adverse socioeconomic conditions, and these problems increased during the years of austerity [3]. So it is possible that health service use may be limited more in those subjects with lower socioeconomic positions.

Research on the relationship of austerity policies with health and with the use of health services is scarce. For example, at the end of 2020, only three works on austerity and mortality policies had been published in the population [2, 4, 5]. The results did not show a clear relationship. And a study carried out in Spain found no relationship between the reduction in the number of health workers and in the number of hospital beds with mortality in the population, or with the rate of hospital readmission or with in-hospital mortality [6]. On the other hand, the introduction of copayment was associated with an increase in the average length of hospital stay [6]. The absence of empirical evidence impedes establishing if austerity policies reduced the use of health services, and, indeed, whether there was any effect on equity in health service use, that is, equality in the use of health services for the same level of care need [7]. The variety of measures implemented across Europe offers the opportunity to answer this question by analysing what occurred in a country which introduced austerity measures, and another which did not for health spending. These two countries are Spain and Germany. Spain was one of the European countries in which very strict austerity measures were implemented [1]. Specifically, austerity measures were put into effect in Spain from the second quarter of 2010 until 2014, although some of the deepest cuts ended in 2013 [8]. One of the measures implemented by the Spanish government was to reduce public spending on the health service [1, 9], while in Germany the health system was protected from austerity measures [1]. In Spain the mean annual growth in health spending was − 3.3% between 2010 and 2013, while the same figure for Germany was 2.6% [10]. From 2014, Spain’s public health spending returned to positive growth, with an average between 2014 and 2017 of 3.5%, although this was still below Germany’s 4.3% [10]. In Spain, the upward trend in the number of professionals and in other health resources, such as hospital beds, observed before the austerity period, turned downward during the austerity years [11]. In addition, in 2012 the Spanish government modified the co-payment system for medicines, making it means tested, although for the elderly a monthly limit of between 8 and 60 euro was set, depending on income [1, 12]. A means test determines if a person is eligible to receive some sort of benefit, such as the reduction in the cost of medicines. If this reduction does not occur, some people may forgo medical consultations, since, predictably, the result of the medical consultation is the prescription of some medicine.

This paper aims to estimate the frequency of doctor’s visits and hospitalizations, and their association with socioeconomic position; this analysis was undertaken using data from the Spanish population both during and after austerity, and, secondly with German population data. Given that the principle of equity in the welfare state is to achieve equal use of healthcare for the same healthcare need, we made estimations for subjects both with and without diseases. Based on the previous arguments, the hypothesis of our study was the probable existence in Spain, but not in Germany, of a decrease in the frequency of use of health services in subjects with diseases, especially in those with a lower socioeconomic position. Therefore, the magnitude of the association of socioeconomic position with the frequency of use of health services will be different in Spain than in Germany.

## Methods

### Data sources

The Spanish data were taken from the 2009 and 2014 European Health Surveys in Spain and from the 2011 and 2017 National Health Surveys carried out by the Ministry of Health and the Spanish Statistical Office. In both, the European and the National Surveys, the sampling framework used the Spanish non-institutionalized population aged 16 and over. The survey had a two-stage sample design. The first-stage units were census tracts, and the second-stage units were the households in each of the selected tracts. The households were selected by simple random selection, and one adult aged 16 or over was selected within each household. A detailed account of the National Health Survey and its data structure can be found on the websites of the Spanish Statistical Office and Ministry of Health [13, 14].

The data for Germany were taken from the 2009, 2011, 2015 and 2017 Socio-Economic Panels (SOEP). The SOEP is a nationwide longitudinal survey project hosted by the German Institute for Economic Research, which employs a two-stage stratified sampling design. The regional units of the first sampling stage correspond largely to the electoral districts for the German parliament from which households are drawn. A random route sampling point (voting district) was used to select the households. Within each household, all adults aged 16 or over were selected. The first survey was carried out in 1984, and regular follow-ups are conducted to keep up with recent developments. Panel attrition is compensated for by sampling new subjects each year in order to obtain a sufficiently large number of cases and to avoid bias in respondent composition. A detailed account of the SOEP and its data structure can be found in Haisken-DeNew et al. [15].

### Study variables

The health services investigated were physician’s consultations and hospitalization in each country. In the Spanish surveys, respondents were interviewed about the frequency of their physician’s visits, and had to choose between one of the following four alternatives: less than 4 weeks ago, between 4 weeks and one year, more than a year ago, and never. People were considered to have consulted a physician if they had made any type of consultation during the last 4 weeks. Those who answered yes were asked about the number of consultations made. In the SOEP those interviewed were asked if they had visited a physician in the last 3 months. People were considered to have consulted a physician if they had made any type of consultation in the previous 3 months. Those who answered in the affirmative were asked about the number of consultations made. Then, for each individual we estimated the number of consultations per year. For this, the number of consultations was multiplied by 4 in Germany and by 13 in Spain. In both the Spanish and German surveys, respondents were asked if they had been in hospital overnight during the previous year. Those who answered in the affirmative were considered to have been hospitalized.

The measure of socioeconomic position was education. Education refers to the highest level of education completed by the respondent. The Spanish surveys used the International Standard Classification of Education (ISCED). Subjects were sorted into the following three categories: high (tertiary education, codes 5 and higher of ISCED), medium (secondary education, codes 2, 3 and 4), and low (elementary education, code 1). In the German surveys several education classifications were used. In the present study we used the Comparative Analysis of Social Mobility in Industrial Nations (CASMIN) classification of education. Subjects were grouped into the following three categories: high (tertiary education, codes 3a and 3b in CASMIN), medium (secondary education (codes 2a, 2b, and 2c) and low (elementary education, codes 1a, 1b and 1c).

In both surveys subjects were shown a list of various diseases and were asked whether a physician had ever told them they suffered from any of them; respondents replied yes or no for each disease. Both lists included heart disease, hypertension, diabetes, and mental health illness. From the response to the presence or absence of these diseases, a variable was created that reflected the number of diseases suffered, whose value ranged from zero to four. Likewise, subjects were grouped into three categories: those who presented one or more of the following chronic diseases: heart disease, hypertension, and diabetes; those who presented with mental health illness; and those who did not suffer any of these four diseases.

Sex, age, and type of health coverage were used in the analyses to control for confounding. In both the Spanish and German surveys, respondents were asked what type of health coverage they had. The responses were grouped into three categories: those who had only some type of public healthcare coverage, those who had both public and private health insurance and those individuals who had only private insurance.

### Statistical analysis

For each country and year the analyses were done separately in those three groups of subjects. First, we calculated the age-adjusted number of doctor’s consultations and the age-adjusted percentage of respondents who had been hospitalized. Weights for age standardisation came from the 2013 European Standard Population. We then estimated the magnitude of the relationship between education and the two measures of the frequency of use of health services. With respect to the number of doctor’s consultations, we estimated the average difference in the number of consultations using covariance analysis. Regarding the percentage of respondents who had been hospitalized, we calculated the percentage ratio estimated by binomial regression. In both cases, we used subjects from the high education category as the reference group. The variables included in the models as possible confounders were already mentioned above. The models that focused on those who presented at least one of these diseases also included the number of diseases reported by each subject as confounders.

## Results

Table 1 shows the distribution by characteristics of subjects with heart disease, hypertension or diabetes and subjects without heart disease, hypertension, or diabetes, and of subjects with mental illness, for each of the years studied in both Spain and Germany. Subjects with a chronic illness had the highest proportion of people aged 65 and over; those with mental illnesses had the highest percentage of women; finally, the group without such illnesses presented the lowest percentage of people with primary studies or below.

**Table 1:**
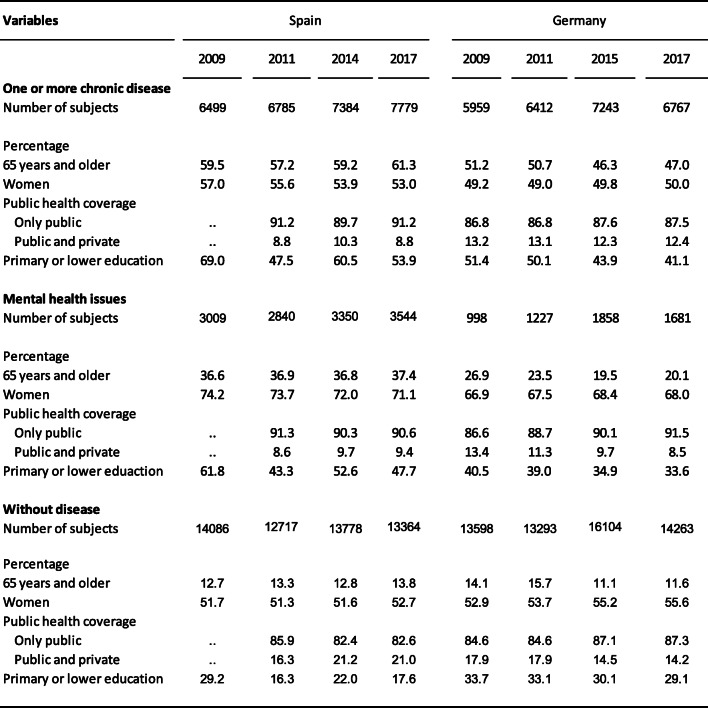
Percentage distribution of population according to different categories of analysis variables. Spain and Germany, 2009, 2011, 2014/2015 and 2017

**Fig. 1:**
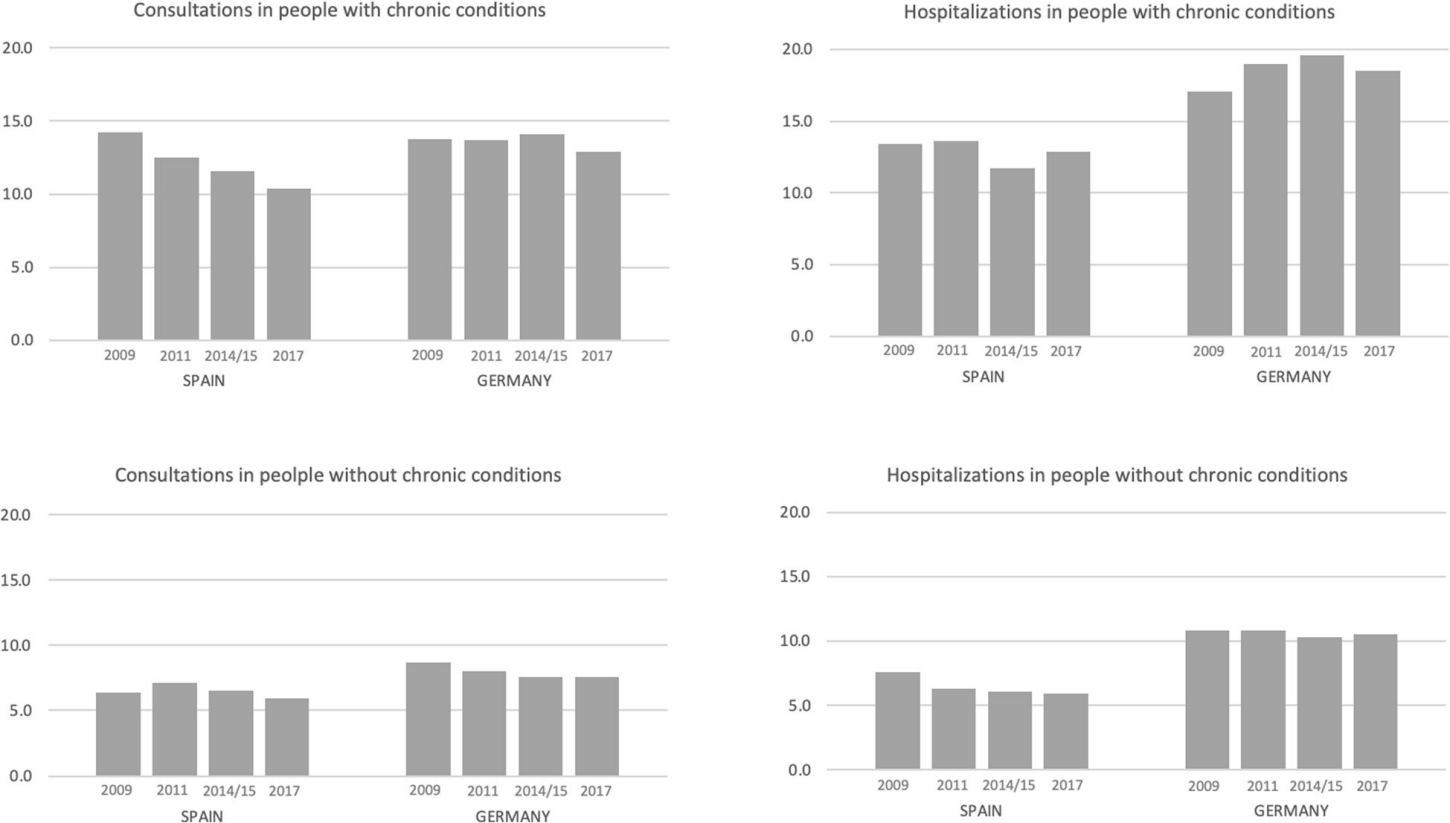
Age-adjusted average annual consultations to doctor and age-adjusted percentage of people who have had at least one hospital admissions in the last year. Spain and Germany, 2009,2001,2014/15 and 2017

Figure 1 and Table 2 shows the annual average – adjusted by age – of doctor’s visits, and the age- adjusted percentage of those who were hospitalized, for each of the years studied in both Spain and Germany. The average number of doctor’s consultations presented a downward trend in both countries. In Spain, average annual consultations were 14.2 in 2009 and 10.4 in 2017 for those with a chronic condition; 16.6 and 13.5 for those with a mental illness; and 6.4 and 5.9 for those without reported diseases. In Germany, average annual consultations were 13.8 in 2009 and 12.9 in 2017 for subjects with a chronic disease; for subjects with a mental illness, 21.1 and 17.0; and 8.7 and 7.6 for subjects without a reported disease. In Spain, the percentage of hospital stays showed a downward trend in all three analysed groups; however, the percentage for those with a chronic disease was actually higher in 2017 than in 2014. In Germany, the percentage of hospital stays for those with chronic diseases and those with mental illness showed an increase between 2009 and 2015, but in 2017 the percentage was lower than 2014. The percentage of hospital stays for those without known diseases was similar across the 4 time periods studied.

s

**Table 2:**
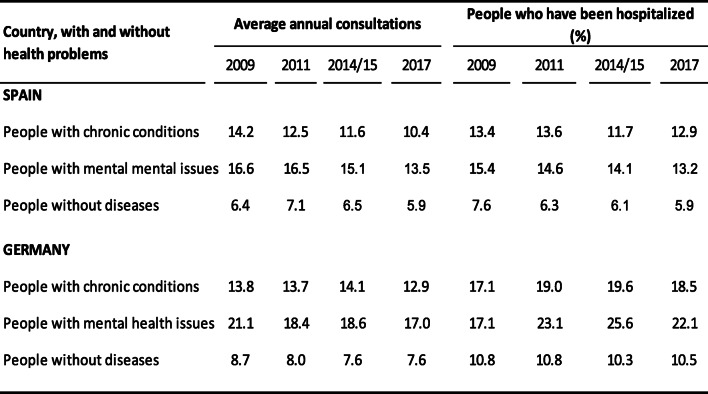
Age-adjusted average annual consultations to doctor and age-adjusted percentage of people who have had at least one hospital admissions in the last year, according to health problems. Spain and Germany, 2009,2001,2014/15 and 2017

Table 3 shows the differences in annual mean doctor’s visits, according to educational level, adjusted by age, sex, healthcare coverage type and number of diseases; in the three groups studied, in both Spain and Germany. In Spain, there were no significant observable differences – except for people with lower levels of studies and a chronic disease in 2011, whose difference with respect to those with higher level studies was 7.7 (95% CI 2.2–12.4)]. The majority of differences in Germany did not show statistically significant differences either – with the exception of lower education levels in those with chronic diseases in 2009 and 2015; the difference was 1.5 in 2009, (95% CI 0.1–2.9) and 1.6 in 2015, (95% CI 0.2–2.9).

**Table 3:**
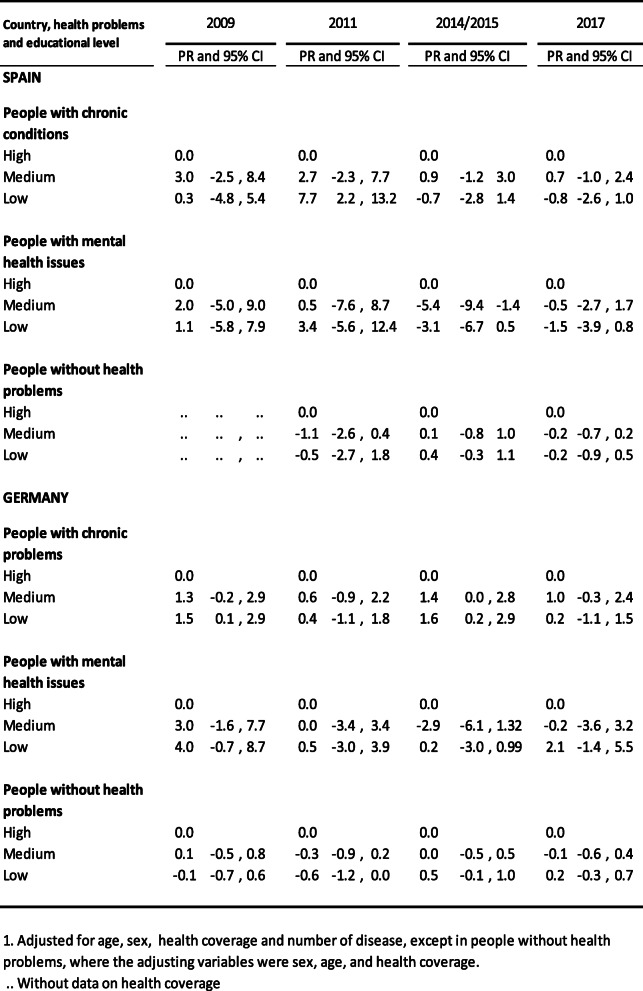
Annual doctor’s consultations with or without health problems, according to educational level. Average difference (AD)1 and 95% confidence intervals (CI) Spain and Germany, 2009,2011,2014/15 and 2017

Table 4 shows the percentage ratios for hospital stays according to educational level, adjusted for age, sex, healthcare coverage type and number of diseases, in the three groups analysed, in both Spain and Germany. There were no significant differences found in Spain, with the exception of those with a chronic disease in 2009; the percentage ratio in relation to those with higher level studies was 0.75 (95% CI 0.67–0.95). No significant differences were found in Germany for this variable either. The exception in this case was subjects with a chronic illness in 2011 and 2015, for whom the percentage ratio was 1.59 in 2011 (95%CI 1.18–2.15) and 0.82 in 2015 (95%CI 0.67–0.99).

**Table 4:**
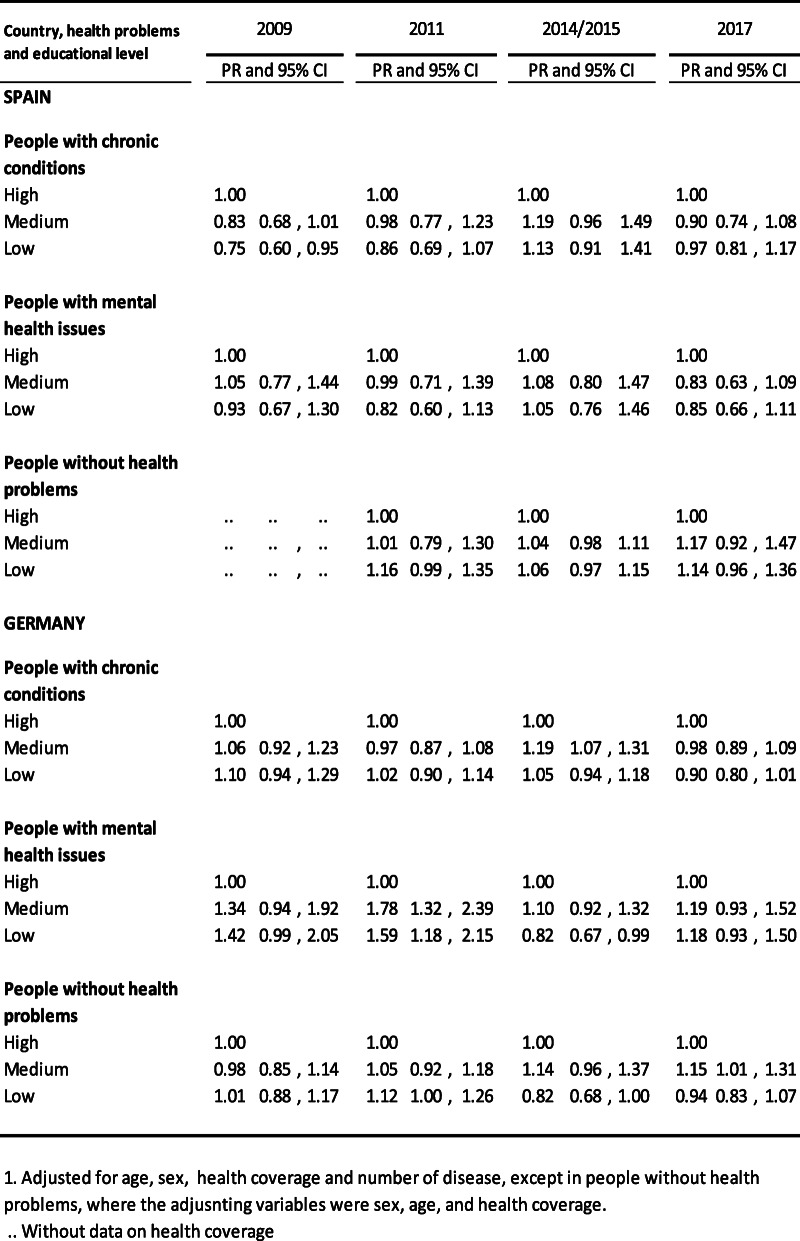
People who had at least one hospital admission in the previous year according to education level. Percentage ratio (PR)1 and 95% confidence interval (CI) Spain and Germany, 2009,2011,2014/15 and 2017

## Discussion

### Principal findings

Between 2009 and 2017, annual mean doctor’s visits for subjects with a chronic disease, those with mental illness, and those without reported diseases showed a downward trend in both Spain and Germany. In Spain, the number of people who had spent time in hospital also showed a downward trend, while in Germany this presented an initial increase followed by a subsequent decrease in subjects with an illness and hardly varied in subjects without diseases. With some exception in low educational level in 2011 in Spain and 2009 and 2015 in Germany, there were no significant differences in mean doctor’s visits nor in hospitalizations in relation to education level in either Spain or Germany – once potential confounders had been controlled for.

### Comparison with other studies and possible explanations

According to some authors, austerity policies have a negative psychological impact on citizens, especially people with mental illnesses [16]. This would be expected to lead to an increase in medical consultations. This negative psychological impact apparently did not occur in Spain; the percentage of the population with poor mental health – according to the 12 Question General Health Questionnaire (GHQ-12) [17] – actually went down in the period studied [18]. In addition, our findings show that the number of consultations decreased in all of the groups studied.

Probably, there are some unknown factors apart from a direct need for healthcare that are associated with doctor’s consultations, for both sick and healthy subjects. That the number of doctor’s consultations decreased during the period analysed – both in subjects with and without the diseases under study – suggests that these factors must have decreased in both Spain and Germany. On the other hand, it is not possible to attribute the decrease in the number of consultations in Spain to austerity measures, as this trend continued after the end of austerity and the number of consultations was still lower in 2017 than in 2014. It is important to note that this trend was also seen in Germany, where health care costs are covered by insurance premiums of citizens without direct state intervention.

Some authors have suggested that a decrease in the number of doctor’s consultations could be associated with an increase in hospitalization frequency, as the health situations of those with diseases would get worse over the medium and long term [19]. This argument, made in the context of considerations about the possible effects of the austerity measures implemented in Spain, is not supported by our findings; in fact the percentage of people who had to be admitted to hospital presented a downward trend. In any case, it is not possible to demonstrate a correlation between the trend in the number of doctor’s consultations and hospitalization frequency – as the results from Germany across the study period show. In Germany, the downward trend in the number of consultations was coincided with an increase in hospitalizations at the start of the study period, but with a reduction in hospitalizations at the end.

Our findings show that it appears that the downward trend in frequency of the use of health services in both Spain and Germany has not altered the balance of equity in healthcare. With some exceptions, no significant differences were observed in the use of health services according to education level, either in subjects with the diseases studied or those without. The fact that the socioeconomic pattern in health services is similar in subjects both with and without diseases shows that unknown factors related to health service utilization were associated with a downward trend across all studied groups. For example, an improvement in clinical practice should not be ruled out as an explanation for the similar findings observed in both countries.

This absence of evidence for inequalities in health service use confirms Eurostat’s findings about the percentage of the population with self-reported unmet needs for medical care. During the austerity years in Spain that percentage – around 6% – barely changed, and had decreased to a figure below 1% by 2017 [20, 21]. Similar figures were found for Germany. Likewise, socioeconomic differences in percentage of self-reported unmet needs for medical care were non-existent in Spain during the years analysed [20]. In Germany, socioeconomic differences in that percentage were small in 2009 and 2011 and had disappeared in 2015 and 2017 [20, 22].

Future studies should examine why the percentage of citizens with self-reported unmet needs for medical care has been decreasing, at the same time that healthcare service use has also decreased. The fact that this decrease in the frequency of healthcare service use has occurred in subjects both with and without chronic diseases suggests that these reasons are not directly related to the need for healthcare. Furthermore, that this decrease has been noted both in Spain and Germany – two countries, where the evolution of the healthcare budget has been different – suggests that these reasons are not related to the supply of healthcare resources either. These findings raise numerous uncertainties about the possible effect that austerity in the healthcare system has on the use of healthcare services. Research on that matter is practically non-existent. Given that our findings come from only two countries, similar research is needed in other countries of the Welfare State, both in those with austerity policies in the health system and in those without such policies. In the case of obtaining the same findings as those observed in Spain and Germany, the prioritization in the allocation of public resources could be reconsidered and directed to other dimensions of equity instead of the health system.

### Strengths and limitations

Evaluating equity in the use of healthcare services requires estimate equality in the use of healthcare services in relation to the need for health care [23]. Subjective health perception has classically been used as measure of the healthcare need. However, that measure may reflect other social necessities, rather than just a need for health care. In our study, we have used an alternative approach by analysing the use of health services, both by those subjects with chronic health problems and those who did not present such conditions. Furthermore, future studies must consider multimorbidity as a healthcare need [24, 25]. For this reason, in our analysis of subjects with health problems we have adjusted for the number of diseases reported by each subject.

We have found some differences in the data sources from Spain and Germany, such as the different year of some of the surveys, the different surveys and designs, and extrapolating the number of physician visits from 4 weeks versus 3 months. This can be a limitation when comparing the estimates of one country with those of the other. However, the methodology of the data source used in each country was the same, which allows an adequate comparison of the estimates within each country in the different studied years.

Our findings are based on self-reported data about doctor’s visits, and as such there is a risk of recall bias. This bias generally occurs with relatively long periods, like the 12 months prior to interview [26, 27]. However, we do not think that this bias would have affected the trends found in this study, as it is highly unlikely that recall bias would vary from 1 year to the next.

## Conclusions

Our findings reflect a decrease in the frequency of health service use between 2009 and 2017 in Spain and in Germany, both in subjects with health problems and in those without. During the first few years, this decrease in Spain coincided with the implementation of austerity measures. Furthermore, across the years studied, there were no observable socioeconomic differences across all of the groups of subjects studied. Given that these are two countries with universal health care coverage and in one of them the austerity policies were intense, these findings suggest that in the distribution of public resources, during periods of austerity, other dimensions of equity should probably be prioritized rather than healthcare.

## Data Availability

The data of Spain can be obtained freely in the web of the Ministry of Health Social Affairs and Equality: https://www.msssi.gob.es/estadEstudios/estadisticas/solicitud.htm. The data of the German Socio-Economic Panel (SOEP) are available for scientific purposes for academic users within and outside of Germany. They can be obtained on request by the dataholder, the Deutsches Institute of Economic Research (Deutsches Institut für Wirtschaftsforschung- DIW). Information and documentations can be found on http://www.diw.de/soep
